# Interaction of Hyaluronan Acid with Some Proteins in Aqueous Solution as Studied by NMR

**DOI:** 10.3390/membranes13040436

**Published:** 2023-04-15

**Authors:** Daria Melnikova, Catherine Khisravashirova, Tatiana Smotrina, Vladimir Skirda

**Affiliations:** 1 Department of Molecular Physics, Institute of Physics, Kazan Federal University, Kazan 420011, Russia; 2 Department of Chemistry, Mari State University, Yoshkar-Ola 424002, Russia

**Keywords:** diffusion coefficient, globular protein, protein, translational diffusion, polysaccharide

## Abstract

According to actual literature data, hyaluronic acid (HA) that is presented in the extracellular matrix can interact with proteins and thereby affect several important functions of the cell membrane. The purpose of this work was to reveal the features of the interaction of HA with proteins using the PFG NMR method by sampling two systems: aqueous solutions of HA with bovine serum albumin (BSA) and aqueous solutions of HA with hen egg-white lysozyme (HEWL). It was found that the presence of BSA in the HA aqueous solution initiates a certain additional mechanism; as a result, the population of HA molecules in the gel structure increases to almost 100%. At the same time, for an aqueous solution of HA/HEWL, even in the range of low (0.01–0.2%) HEWL contents, strong signs of degradation (depolymerization) of some HA macromolecules were observed such that they lost the ability to form a gel. Moreover, lysozyme molecules form a strong complex with degraded HA molecules and lose their enzymatic function. Thus, the presence of HA molecules in the intercellular matrix, as well as in the state associated with the surface of the cell membrane, can, in addition to the known ones, perform one more important function: the function of protecting the cell membrane from the destructive action of lysozymes. The obtained results are important for understanding the mechanism and features of the interaction of extracellular matrix glycosaminoglycan with cell membrane proteins.

## 1. Introduction

The plasma membrane of mammalian cells is a highly dynamic structure, whose biomechanical properties play a vital role in the regulation of many functions of the living cell, such as adhesion, migration, signal transmission and others [1]. It is believed that one of the most dynamic processes within these membranes is the formation of fine structures, which, in turn, are involved in intercellular adhesion [2]. There are works that experimentally show that it is these fine structures that provide the pathway for intracellular and intercellular communication [3,4,5].

Many molecular components, including various proteins, may be involved in the formation of intercellular (intermembrane) connections, which, in turn, may affect the characteristics of cell membranes themselves. Among the various components of the extracellular matrix, polysaccharides (glycosaminoglycans) play an important role, as they have the greatest variability and represent the most dynamic structures in tissues. Many enzymes are known to specifically “adapt” proteoglycan molecules during pathophysiological processes [6]. For example, the tumor necrosis factor, alpha-stimulated protein TSG6, can covalently bind to HAs, which in turn promotes the transfer of the inter-alpha trypsin inhibitor chain to the COOH bonds of HAs. As a result, this transfer leads to the formation of a complex called serum hyaluronan-associated protein (SHAP), which is involved in many pathologies [7]. It follows from the works of [7,8] that protein complexes with HA have the ability to change not only the local properties of the membrane itself, but also, acting as an external cytoskeleton, to modify and control the shape of the cell.

Thus, HA molecules are an integral component of both the extracellular and pericellular matrix (i.e., in direct contact with the membrane surface), where they interact with it through membrane proteins [9].

The purpose of this study is to try to establish additional effects of the interaction of HAs with some proteins based on translational mobility, which is quite sensitive [10] to the formation of various intermolecular bonds. As candidates for studying their interaction with HA, in this work, we will focus on the previously well studied [11] PFG NMR bovine serum albumin (BSA) and lysozyme (HEWL) methods.

The peculiarity of the BSA protein is that this protein belongs to the category of receptor proteins. BSA is characterized by the ability to form complexes with various organic and inorganic ligands due to the existence of many different binding centers [12,13]. Thus, BSA can perform the function of transporting physiological metabolites and some drugs [14,15]. In the “BSA + HA + Water” system, the molecular motion of the globular BSA protein can be affected [11] by intermolecular interactions with hyaluronic acid. Moreover, this refers to the interaction of BSA with a gel network [16,17,18] formed by HA molecules. At the same time, in an aqueous solution of HA, a gel structure is formed due to hydrogen bonds between macromolecular chains [19,20,21].

Compared to BSA, HEWL is more “active”. It is a relatively small protein with a molecular weight of 14.3 kDa and an isoelectric point of 11.35, which makes it cationic (total charge + 7) at neutral pH [22]. It makes sense to note that this protein belongs to the category of enzymatic proteins. Thus, according to the previous research [23,24], this lysozyme is capable of cleaving the glycosidic bonds of polysaccharides due to the asparagine and glutamic acids present in its amino acid sequence. The same protein can perform antibacterial functions [25], penetrating inside the bacterial cell and destroying the cell membrane.

Thus, this work will present the results of the study of the features of the interaction of HAs with two proteins that are quite different in their characteristics (BSA, HEWL). At the same time, all three are quite important components of the intercellular (intermembrane) matrix. Of fundamental importance is also the fact that the HA molecule is an integral component of not only the extracellular but also the pericellular matrix [26,27,28], where it interacts directly with the membrane surface through, for example, the membrane protein glycoprotein CD 44 [28,29,30].

## 2. Materials and Methods

Pure hyaluronic acid (HA; 3000 kDa) was provided by the Department of Chemistry, Mari State University. Bovine serum albumin (BSA; 66 kDa; pH 7, ≥98%) and hen egg-white lysozyme (HEWL; 14.3 kDa; lyophilized powder, protein ≥ 90%, ≥40,000 units/mg protein) were obtained from Sigma-Aldrich (St. Louis, MO, USA). Deuterated water (D_2_O; 99.9%) was purchased from Deutero GmbH (Kastellaun, Germany). All materials were used without further purification.

All NMR measurements were performed at 298 K on a 400 MHz Bruker Avance-III TM spectrometer equipped with a gradient system that allowed for a maximum gradient, g, of 28 T/m (e.g., 2800 G/cm). Temperature was calibrated using a set of test samples with known diffusion coefficients. Self-diffusion coefficients (hereinafter referred to simply as diffusion coefficients) were measured using the stimulated-echo pulse sequence (PGSTE) [31]. ^1^H experiments were performed using 48 different values of g, a gradient pulse duration δ of 1 ms, the time between the leading edges of gradient pulses Δ = 50 and 300 ms, the time interval between the first and the second radiofrequency pulses τ ranging from 6 ms, and a recycle delay of 15,000 ms.

The measurement of self-diffusion coefficients of molecules by NMR is based on registration of loss-of-phase coherence of the molecules’ spins due to spatial displacements of molecules in the magnetic field gradient [32]. If the translational mobility of molecules is not limited, the distribution of their spatial displacements is described by a Gaussian function:

(1) Psr−,r−′,t=14πDst32exp−r−′−r−24Dst,

where $P_{s}\left(\overset{-}{r},{\overset{-}{r}}^{\prime },t\right)$ is the conditional probability density or “propagator” of spin detection at the radius vector ${\overset{-}{r}}^{\prime }$ at time t, if at the initial time point the spin was at the radius vector $\overset{-}{r}$; $D_{s}$—self-diffusion coefficient (SDC) of molecules.

The primary information in the PFG NMR method was obtained from the analysis of the diffusion decay $A\left(\tau_{1},\tau_{2},g,t\right)$—dependence of the spin echo signal amplitude on the magnetic field gradient parameters and time t. For the stimulated echo sequence, the decay of the signal amplitude was determined by the expression:

(2) τ1,τ2,g=A02exp−2τ1T2−τ2T1exp−γ2g2δ2∆−δ3Ds,

where $A_{0}$—initial amplitude of the echo signal, $\tau_{1}$ and $\tau_{2}$—time intervals between the first and second, second and third RF pulses, respectively, $T_{1}$ and $T_{2}$—nuclear magnetic relaxation times, γ—gyromagnetic ratio of protons, δ and g—duration and amplitude of magnetic field gradient pulses, ∆—time interval between two successive gradient pulses, the expression ∆−δ3 is diffusion time $t_{d}$.

A direct determination of the self-diffusion coefficient of molecules can be made from the determination of the tangent of the envelope of the echo amplitude (diffusion decay), which has the form of a straight line in coordinates $lg\left(A\left(g^{2}\right)/A\left(0\right)\right)/\gamma^{2}g^{2}\delta^{2}\cdot t_{d}$. In the case of a nonexponential form of diffusion decay, we can analytically describe the decay of the amplitude of the echo signal by an expression of the form:

(3) $$A\left(\tau_{1},\tau_{2},g\right)=A_{0}\sum_{i}^{N}p_{i}\exp\left(-\gamma^{2}g^{2}\delta^{2}D_{si}t_{d}\right),$$

where $p_{i}$ is the “weight” coefficient of the *i*-th exponent, characterized by the effective self-diffusion coefficient $D_{si}$. The NMR method with a pulsed magnetic field gradient makes it possible to measure the SDC of molecules in the range of $10^{-8}÷10^{-15}m^{2}/s$.

## 3. Results and Discussion

### 3.1. Interaction of Bovine Serum Albumin with Hyaluronic Acid Molecules

**Figure 1:**
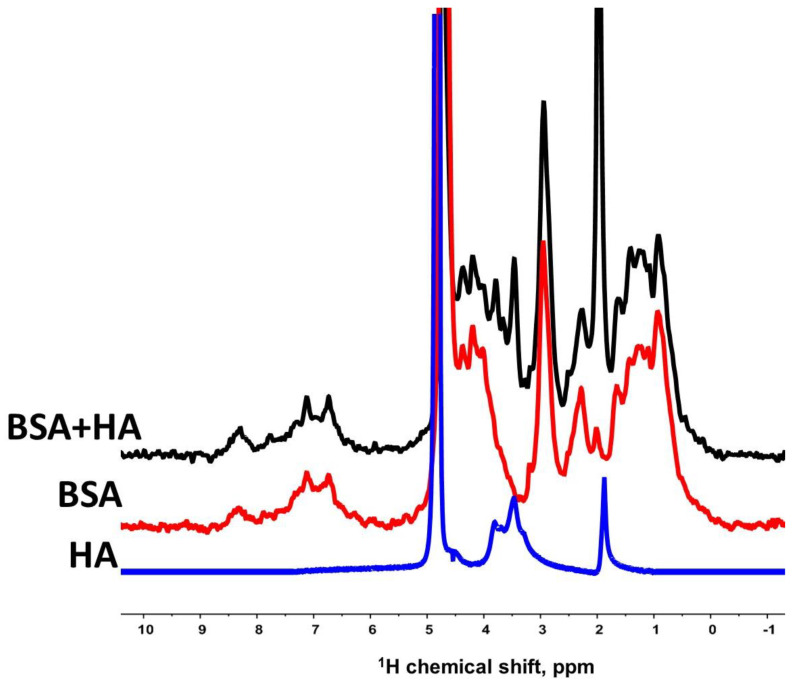
Proton NMR spectra of certain samples. BSA + HA—solution of albumin and HA at a protein concentration of 4% (wt.) and 0.75% hyaluronate; BSA—solution of BSA at a concentration of proteins 4% (wt.); HA—aqueous solution of HA with a polysaccharide concentration of 0.75% (wt.). Spectral data were recorded at 298 K.

When researching the translational mobility of each molecular component of the “BSA + HA + Water” system by NMR methods with PFG in a spectrally resolved mode for the further isolation of characteristic signals of protein and polysaccharide molecules and obtaining characteristic spin echo signals for them, NMR proton spectra of the studied system and water solutions of the initial components of BSA and HA were recorded (Figure 1).

In the proton NMR spectrum of the BSA solution, the signal characteristics of the protein are in a fairly wide range of chemical shifts: according to [33], the signals in the range of chemical shifts characteristic of aliphatic groups in the range of 3.1–2.8 ppm are due to the presence of Cys34 (C_β_H_2_ groups); signals in the region of 2.08–1.98 ppm are attributed to the protons of such amino acids as glutamine Gln33 (signal of the C_γ_H_2_) and proline Pro35 (signal of the C_γ_H_2_); signals in the intervals of chem. shifts of 8.2–7.5 and 7.1–6.9 ppm should be attributed to the signals from the C_ε_H and C_γ_H_2_ groups of histidines, respectively. The region at 7.3–6.6 ppm corresponds to the signals of aromatic rings of tyrosine residues [34].

**Figure 2:**
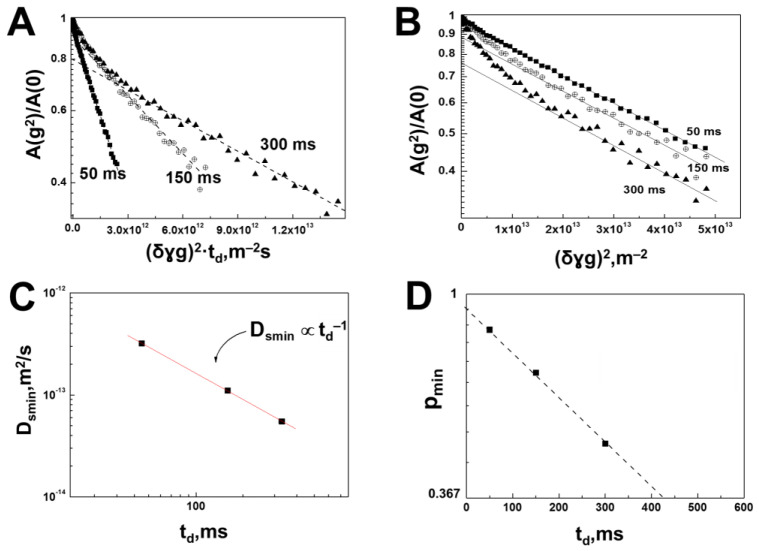
Data for an aqueous solution of HA at a polysaccharide concentration of 0.75%. (**A**) Diffusion decays of the spin echo signal of HA molecules obtained at diffusion times of 50, 150 and 300 ms. The lines show the diffusion decay components characterized by the minimum SDC. (**B**) Diffusion decays of the spin echo signal of HA molecules obtained at diffusion times of 50, 150 and 300 ms plotted in coordinates without $t_{d}$. The lines show the diffusion decay components characterized by the minimum SDC. (**C**) Dependence of the minimum SDC of HA molecules on the diffusion time. The solid line is the slope of $t_{d}^{-1}$ and shows that experimental values of $D_{s}$ are inversely proportional to $t_{d}$. (**D**) Population $p_{min}$ (solid squares) with minimum SDC are plotted as functions of diffusion time molecules.

The recorded diffusion decays of the spin echo signal for HA molecules in a water solution at a polysaccharide concentration of 0.75% (wt.) have a complex form, while for HA molecules characterized by a minimum SDC of about $\sim 10^{-14}$ m^2^/s, there are clearly signs of limited diffusion. First, some of the HA molecules are characterized by the $D_{smin}$ values, which depend on the diffusion time $t_{d}$. Second, this dependence corresponds to the mode of completely limited diffusion $D_{smin}\propto t_{d}^{-1}$. This follows from the coincidence of the finite slopes of the diffusion decays shown in Figure 2B in the coordinates $lg\left(A\left(g^{2}\right)/A\left(0\right)\right)/\gamma^{2}g^{2}\delta^{2}t_{d}$, as well as from the dependence $D_{smin}(t_{d})$ itself (Figure 2C).

As a result of a comparative analysis of the spectra presented in Figure 1, it can be concluded that the spectrum of an aqueous solution of BSA and HA contains, as expected, signals of both protein and hyaluronate. On the proton spectrum of an aqueous solution of HA, the signal in the region of 1.9 ppm according to [35], it is characteristic of the protons of the methyl (-$CH_{3}$) *N*-acetyl group of hyaluronate. Signals located in the region between 3.8 and 3.0 ppm correspond to signals from protons of disaccharide units of HA. Thus, when studying the “BSA + HA + Water” system, we could obtain data on the translational mobility of both protein and HA molecules.

In systems such as gelatin [36], this phenomenon is due to the formation of a supramolecular gel structure. Thus, the dependence of SDC on diffusion time, shown in Figure 2C, allows us to make the conclusion that HA molecules in a water solution at a concentration of 0.75 (wt.) form a supramolecular structure—a three-dimensional gel network. This state of HA molecules means that the root-mean-square (rms) displacement remains constant since $\langle r^{2}\rangle \sim t_{d}^{0}$, as follows from the equation:

(4) $$D=\frac{\langle r^{2}\rangle }{6\cdot t_{d}}\propto t_{d}^{-1}.$$

Estimation of the restriction size or gel grid size formed by HA molecules by Formula (4) gives the value $\langle r^{2}\rangle =0.314\pm 0.016$ μm. The second important result is that the fraction of HA molecules characterized by the sign of completely restricted diffusion (Expression (4)) depends on the diffusion time. This is demonstrated from the comparison of the curves shown in Figure 2B. Figure 2D directly shows the population $p_{min}$ dependence on $t_{d}$.

It can be supposed that such a change in the population of the gel component is associated with the manifestation of the lability of the gel formed by HA molecules. Such behavior, namely, limitation of diffusion of macromolecules in meshes formed by natural polymers, along with changes in the population of molecules involved in the formation of supramolecular structures, has already been observed [10,37].

The dependence shown in Figure 2D can be approximated by the function:

(5) $$\frac{p_{min}\left(t_{d}\right)}{p_{min}\left(0\right)}=exp\left(-\frac{t_{d}}{\langle \tau \rangle }\right).$$

The dotted line shown in Figure 2D corresponds to Expression (5) at values of $\langle \tau \rangle =415\pm 31$ ms and $p_{min}\left(0\right)=0.9\pm 0.03$. Thus, from the results of the study of self-diffusion of HA molecules in an aqueous solution with a HA concentration of 0.75%, it follows that 90% of the HA molecules form a gel network, and 10% are in a free state. The observed dependence $p_{min}\left(t_{d}\right)$ is itself a consequence of the molecular exchange of HA molecules between the free state and the state in the gel net. The obtained value $\langle \tau \rangle$ can be interpreted as the lifetime of HA molecules in the gel state within the given reasoning. The obtained characteristics of the translational mobility of HA molecules in aqueous solution can serve as a certain reference for the study of more complex molecular systems containing additional protein components.

**Figure 3:**
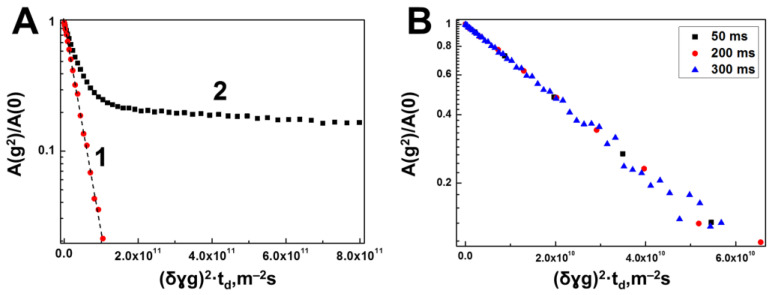
Diffusion decays of the spin echo signal for a mixture of BSA and HA obtained in the spectral resolution mode at a polysaccharide concentration of 0.75% and protein concentration of 4%. (**A**) Diffusion decays obtained at diffusion time of 50 ms by integrating the signals of the proton spectrum of a mixture of BSA and HA for the chemical shift regions from 6.6 to 8.2 ppm (curve 1), specific to BSA molecules, and for the chemical shift region from 1.8 to 3.9 ppm, in which there are signals from both HA and from BSA (curve 2). (**B**) Diffusion decays for BSA molecules at various diffusion times of 50, 200 and 300 ms, obtained by integrating signals in the range of chemical shifts from 6.6 to 8.2 ppm.

Figure 3 shows the diffusion decay of the back echo signal for “BSA + HA + Water” in the spectrally resolved mode.

As a result, we can judge unambiguously enough about the translational mobility of each of the components of the “BSA + HA + Water” system. Translational mobility of BSA molecules in the solution with HAs is characterized by a single self-diffusion coefficient, equal to $3.7\times 10^{-11}m^{2}/s$, which is quite close to the value of the self-diffusion coefficient of freely diffusing protein molecules in the “BSA + Water” solution with the same concentration (4%) of protein, equal to $4.28\times 10^{-11}m^{2}/s$. At the same time, the lack of dependence of the diffusion decay shape of BSA molecules on the diffusion time shown in Figure 3B is more evidence of the unrestricted diffusion of BSA molecules in aqueous HA solution.

In [38], where the translational mobility of BSA in solution with HAs was also studied, a 1.5-times decrease in the BSA SDC was registered compared to the SDC of the protein in water solution at the same concentration. The authors of this work suggest that this effect is a consequence of the formation of complexes between BSA molecules and HAs. In our opinion, such a hypothesis has the right to exist, although, as in [38], it is not possible to register a direct sign of complex formation, specifically, the establishment, at least for some albumin molecules, of the SDC values coinciding with the SDC values of HA molecules. Nevertheless, the observed decrease in the SDC values of BSA molecules in the solution with HA as compared to the aqueous solution of BSA cannot be explained only by the influence of the restrictions of the HA polymer chains due to a too low (0.75%) concentration of HA. Hence, it is reasonable, as well as in [38], to assume the formation of BSA–HA complexes, which, however, are characterized by short lifetimes.

Note that in [38], it was not established in what state the hyaluronate molecules are in the “BSA + HA + Water” system. To determine this state, we needed to obtain and analyze the diffusion decays of the spin echo signal for the HA molecules by integrating the signals located in the region of chemical shifts from 1.8 to 3.8 ppm (the spectrum shown in Figure 1).

**Figure 4:**
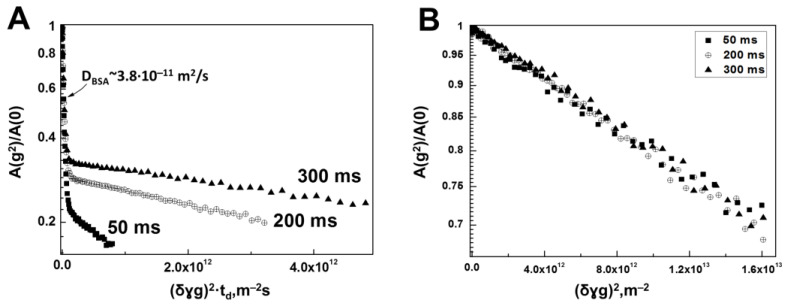
(**A**) Diffusion decays of a water solution of a mixture of HA and BSA at polysaccharide and protein concentrations of 0.75% and 4% (wt.) respectively, obtained by integrating signals located in the range of chemical shifts from 1.8 to 3.8 ppm at various diffusion times of 50, 200 and 300 ms. (**B**) The diffusion decays for HA molecules only, obtained by subtracting the contribution to the signal of the spin echo of BSA molecules and plotted in coordinates as a function of $\gamma^{2}g^{2}\delta^{2}$, demonstrating a restricted diffusion regime.

**Figure 5:**
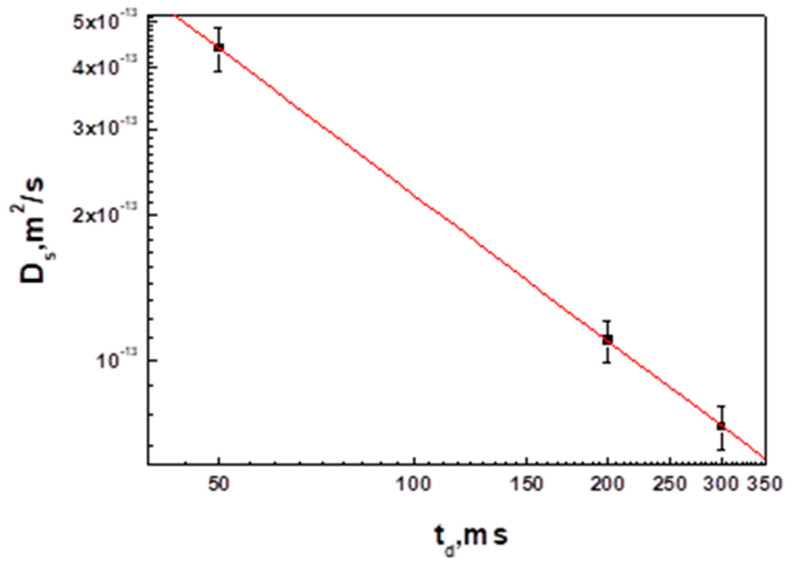
Dependence of the self-diffusion coefficient of HA species $D_{s}$ on diffusion time $t_{d}$. The solid line represents a slope of $t_{d}^{-1}$ and shows that experimental values of $D_{s}$ are inversely proportional to $t_{d}$.

Thus, the obtained dependence of the diffusion decay of the spin echo signal in water solution of HA and BSA (Figure 4A) shows a decrease in the slope of the diffusion decay with increasing diffusion time, which indicates a decrease in the value of the minimum SDC with increasing $t_{d}$ for a part of HA molecules. Figure 4B shows the diffusion decays related only to HA molecules. From this figure, it is well seen that all diffusion decays in the presented coordinates coincide with the accuracy of the experimental error. Figure 5 below shows the dependence of the SDC of HA molecules in water BSA solution on the diffusion time $t_{d}$.

The experimentally obtained dependence of the minimum SDC ${(D}_{s})$ on the diffusion time $t_{d}$ for a sample of water solution of hyaluronic acid 0.75% (wt.) in the presence of 4% BSA indicates that the self-diffusion coefficient $D_{smin}$ is inversely proportional to the diffusion time; consequently, HA molecules in water solution with BSA are in a completely limited state.

The dependence of the self-diffusion coefficient on the diffusion time shown in Figure 5 allows us to conclude that the HA molecules in the presence of BSA in aqueous solution form a supramolecular structure similar to that (gel) in aqueous solutions of HAs. However, the restriction size or gel grid size formed by the HA molecules in the presence of BSA calculated by Formula (5) turned out to be 0.362 ± 0.019 μm, about 14% larger compared to the same value for the sample of aqueous HA solution. Therefore, in an aqueous solution of HAs with BSA, the HA molecules form a gel structure, which is less “rigid” in its characteristics compared to the gel formed in an ordinary aqueous solution of HAs.

However, another experimental fact is more interesting: in the HA system with BSA, we could not find HA molecules with signs of free diffusion. In addition, the independence of the form of diffusion decays (Figure 4B) for HA molecules in the “HA + BSA” system from the diffusion time demonstrates, in contrast to the data shown in Figure 2B, the absence of any signs of molecular exchange. This can be interpreted as the absence of the very “phase” with which such exchange can take place. In other words, it can be argued to the accuracy of the experiment that the presence of the BSA protein in the system initiated some additional mechanism that caused all HA molecules to form a gel structure. At the same time, if there are free HA molecules in the system, their share is negligibly small.

No direct evidence of BSA–HA complex formation could be found, but the occurrence of a decrease in SDC in the BSA molecules in the presence of a rather small (0.75%) amount of HA can be formally interpreted as a consequence of BSA–HA complex formation with a relatively short lifetime. At the same time, the presence of BSA molecules quite noticeably affected the characteristics of the gel structure formed by HA molecules. This result confirms that there is a certain interaction mechanism between BSA molecules and HAs to which characteristics of translational mobility of the high molecular weight component, which is HA, are quite sensitive.

### 3.2. Interaction of Hen Egg-White Lysozyme (HEWL) with Hyaluronic Acid Molecules

Compared to BSA, HEWL is a more “active” protein, as it exhibits antimicrobial activity as a lytic enzyme [39]. It is a protein with a molecular weight of 14.3 kDa and an isoelectric point of 11.35, making it cationic (total charge + 7) at neutral pH [40].

**Figure 6:**
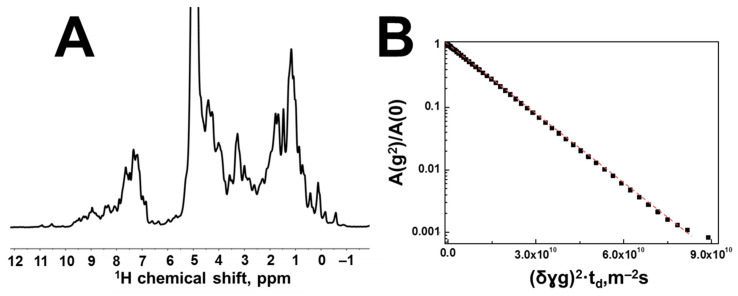
(**A**) Proton spectrum of an aqueous solution of hen egg-white lysozyme at a protein concentration of 4% (wt.). (**B**) Diffusion decays of aqueous solution of HEWL at a concentration of 4% (wt.). Diffusion decay was obtained by integrating the BSA signals located in the region of chemical shifts from 0 to 3 ppm. The solid line shows the SDC with the value $4.28\times 10^{-11}\;m^{2}/s$. This diffusion decay was recorded at a diffusion time of 50 ms and a temperature of 298 K.

A typical view of the proton spectrum of the HEWL solution is shown in Figure 6, in which, similarly to the spectrum of the globular BSA protein solution, one can observe signals in the region of chemical shifts from 7 to 9 ppm.

**Figure 7:**
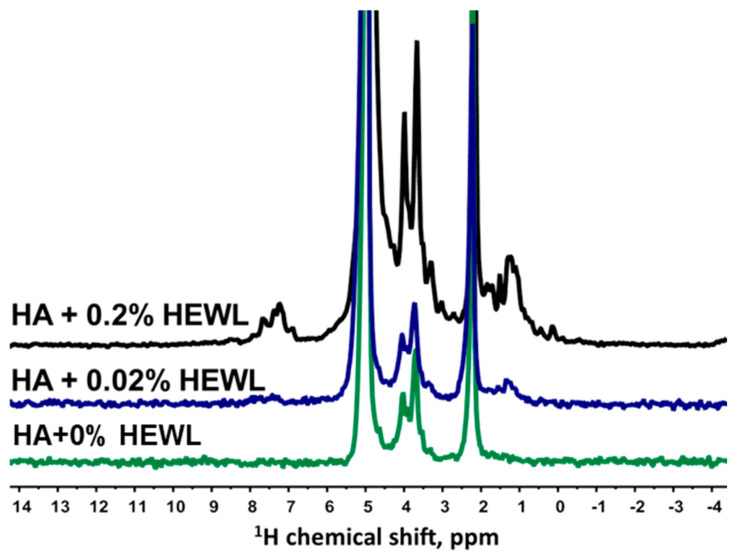
Proton spectra of aqueous solutions of HA and HEWL at a HA concentration equal to 0.75% (wt.) and at different protein concentrations: black color shows the spectrum at a protein concentration of 0.2% (wt.); blue color shows the spectrum at a protein concentration of 0.02% (wt.); green color shows the spectrum of HA aqueous solution.

The translational mobility of HEWL in an aqueous solution at a protein concentration of 4% is characterized by a SDC of $8.6\times 10^{-11}\;m^{2}/s$. Similar to the BSA solution, the shape of the diffusion decay of the spin echo signal of the HEWL solution has a monoexponential shape (Figure 6B), without signs of any processes of association of protein molecules. Since HEWL can specifically interact with polysaccharides [24,41], it makes sense to start the study of aqueous solutions of HA–HEWL with minimal protein concentrations. Figure 7 shows the proton NMR spectra of aqueous solutions of HA with different concentrations of HEWL.

**Figure 8:**
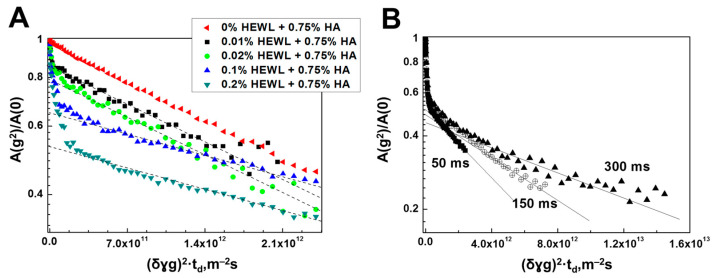
Diffusion decay of the spin echo signal for lysozyme and HA molecules at a HA concentration equal to 0.75% (wt.). (**A**) Diffusion decay at a diffusion time of 50 ms, obtained for samples with different protein contents (from 0 to 0.2 wt.%) by integrating signals located in the chemical shift region from 1.6 to 3.0 ppm. (**B**) Diffusion decays of the spin echo signal for an aqueous solution of HA and HEWL at a protein concentration of 0.2% (wt.) and polysaccharide concentration (0.75%) at various diffusion times of 50, 150 and 300 ms, obtained by integrating the signals of the proton spectrum (Figure 6) in the region of chemical shifts from 1.6 to 3.0 ppm.

In the presented NMR spectra, as expected, with an increase in the concentration of HEWL, characteristic signals of NH groups of the protein appear. Figure 8 shows the diffusion decays of the spin echo signal of HA solutions with different concentrations of HEWL, obtained by integrating signals located in the chemical shift region from 1.6 to 3.0 ppm, which does not contain an intense signal from OH groups.

All diffusion decays of the spin echo signal for aqueous solutions of HEWL and HA, presented in Figure 8, refer mainly to HA molecules, since for the indicated integration region, the signal from HA is dominant compared to the signal from HEWL molecules. As can be seen from Figure 8A, when even a small amount of HEWL is added, global changes in the shape of the diffusion damping of the spin echo signal occur.

Figure 8B shows diffusion decays for various diffusion times $t_{d}$ at 0.2% lysozyme concentration. From these data, one can see that the part of the diffusion decay characterized by minimum self-diffusion coefficient values depends on the diffusion time, and the character of the dependence is similar to that previously found for HA molecules (see Figure 2A and Figure 4A). In this regard, for the indicated part of the diffusion decay showing signs of restricted diffusion, it makes sense to associate it with the presence of the gel structure caused primarily by HA molecules.

**Figure 9:**
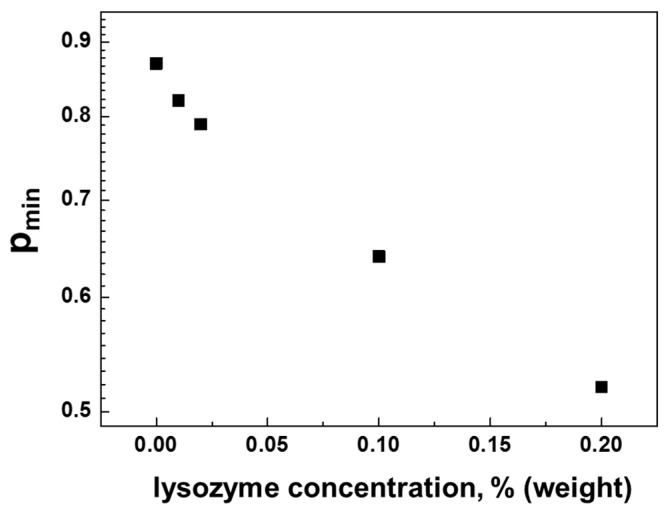
Population of molecules with self-diffusion coefficient having gel features in aqueous solutions of HA (0.75% weight) and HEWL mixtures as a function of protein content. The $p_{min}$ values were obtained from an analysis of the diffusion decays shown in Figure 8A obtained at a diffusion time of 50 ms.

Returning to the discussion of diffusion decay in Figure 8A, we note that the fraction of the signal with signs of restricted diffusion decreases quite clearly with increasing lysozyme content. The dependence of the fraction of molecules with restricted diffusion signs on the lysozyme protein content is shown in Figure 9.

As can be seen from Figure 9, even very low lysozyme protein contents lead to a noticeable decrease in the fraction of HA molecules retaining the ability to form the gel structure. Already at a lysozyme concentration of only 0.2%, the population of such molecules decreases from 90% to about 50%. At the same time, the population of molecules for which there are no signs of gel formation completely increases. Apparently, this is a consequence of the manifestation of the enzymatic properties of the lysozyme. According to [23,24], HEWL is able to cleave the glycosidic bonds of polysaccharides due to the presence of aspartic and glutamic acids in its amino acid sequence.

Attempts to obtain a mixture of HAs and HEWL with more than 0.2% HEWL, for instance, 0.5%, in aqueous solutions produced a translucent/muddy precipitate, which did not disappear with either mechanical/thermal treatment of the sample or attempts to change the acidity (pH) of the aqueous solution. Apparently, this is due precisely to the fact that HEWL is able [42] to form coacervates under certain conditions (LLPS). For example, coacervation of a polyelectrolyte/protein complex is the separation of a solution into two phases due to nonspecific electrostatic interactions [43,44].

At this point of time, coacervates are of considerable interest because they form spontaneously from aqueous mixtures and provide stable compartmentalization without the need for a membrane. In addition, the authors of [45] have illustrated the mechanism of cytoplasm organization arising from clusters of weakly “sticky” molecules, including other assemblies of ribonucleoproteins (e.g., P-bodies, Cajal cells or stress-granules) by the example of a Germline P-granule localization study [46,47]. It was also [45] that suggested that such phase structuring may represent the initial mechanism of functional self-assembly of relatively undeveloped molecular ensembles at the early stages of life evolution.

**Figure 10:**
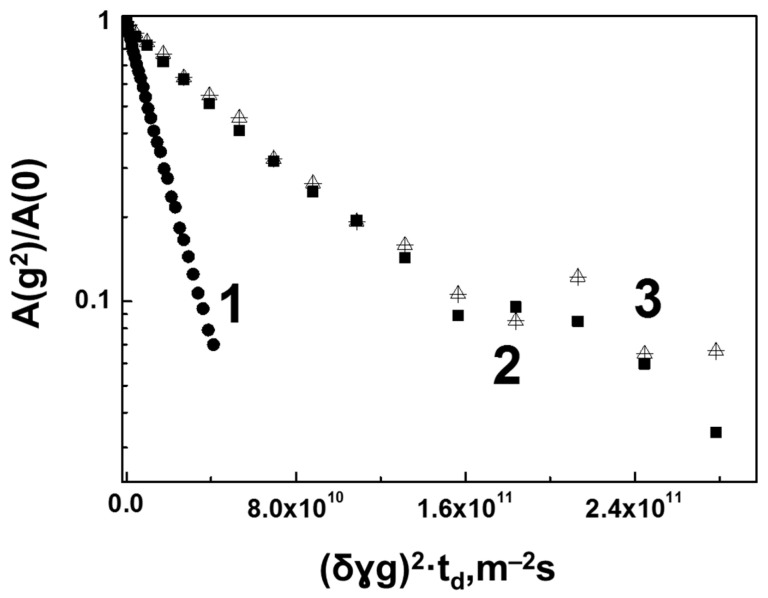
Diffusion decay of the spin echo signal for lysozyme molecules obtained with a diffusion time of 50 ms. Curve 1— lysozyme in aqueous solution at a protein concentration of 0.2%; Curve 2—lysozyme in an aqueous solution of a mixture of lysozyme and HA at concentrations of 0.2% and 0.75% (mass), respectively (obtained by integrating signals in the region of 6.5–8 ppm in the proton spectrum (Figure 7); Curve 3—normalized per unit diffusion decay obtained by subtracting the contribution of molecules with an SDC equal to $1.6\times 10^{-13}m^{2}/s$ from the SDC values for lysozyme and HA aqueous solution at concentrations of 0.2% and 0.75% (weight), respectively, shown in Figure 8. At the indicated integration range (from 1.6 to 3.0 ppm), this diffusion decay mostly refers to the HA.

In total, unlike aqueous solutions of BSA–HA and native solutions of hyaluronate, HA molecules in aqueous solution of the lysozymes undergo severe degradation. Thus, even with a lysozyme content of 0.2%, the proportion of HA molecules retaining the ability to form a gel structure decreases almost twofold. Coacervate formation with increasing protein content cannot be interpreted otherwise than as a consequence of the formation of a complex of HAs with the lysozymes. A similar formation of complexes between proteins and HAs was established earlier for the silk fibroin/HA system [48], as well as for a mixture of HAs and IgG from bovine serum (Bovine IgG) [49]. As an explanation for the formation of phase-separated coacervates, the authors point out the result of weak multivalent interactions between biomacromolecules, despite the understanding of the influence of molecular interactions on the formation and properties of protein–polyelectrolyte coacervates, much remains unexplored. In this context, let us consider our experimental data on the translational mobility of lysozyme molecules in solutions with HAs in more detail. Figure 10 shows diffusion decays for lysozyme molecules in an aqueous solution with a protein concentration of 0.2%, as well as in a mixture with HA at the same protein concentration. For comparison, the same figure shows the diffusion decay for that part of the HA molecules that have degraded and lost signs of limited diffusion.

As can be seen from Figure 10, the diffusion decay (curve 1) for lysozyme molecules in solution with water with a protein concentration of 0.2% is described by an exponential function with a single SDC value, which was found to be $6.9\times 10^{-11}m^{2}/s$. At the same time, in solution with HA at the same (0.2%) concentration of lysozyme, the diffusion decay shape for protein molecules (Curve 2) has a more complex form, and its initial slope is described by a significantly lower value ($1.4\times 10^{-11}m^{2}/s$) of the average SDC. Thus, as compared to the BSA protein, for which a relatively small decrease in the SDC value as a result of interaction with HA was observed; in this case, we see a significant (almost five-fold) decrease in the translational mobility of lysozyme molecules in the presence of HA. Even more interesting is the result of comparing the diffusion decay of lysozyme molecules (curve 2) with the diffusion decay (curve 3) for that part of HA molecules which, as mentioned above, were degraded by the lysozymes and lost their ability to form a gel structure. The indicated diffusion decays coincide with the experimental error. Such coincidence of the translational mobility characteristics for the lysozyme molecules and the degraded part of the HA molecules unambiguously testifies to the formation of a sufficiently strong HEWL–HA complex. In other words, this result suggests that during the interaction of lysozymes with HAs as a result of the cleavage of the glycoside bonds in the HA molecule by the active amino acids of lysozyme [50,51], the lysozyme molecule does not remain free, but is attached in some way to one of the parts of the hydrolyzed HA molecule. In contrast to the common model [51,52], this result agrees with the results of [53] in which the study of aqueous dextran solutions showed that after the reaction of glycoside bond hydrolysis by the lysozymes, covalently bound protein and dextran complexes are found.

In conclusion, the data presented in Figure 9, do not depend on the exposure time of the sample. This fact, combined with the established fact of the formation of a complex between lysozyme molecules and degraded HA molecules, indicates that lysozyme molecules lose their enzymatic activity after interaction with HA. This conclusion allows us to hypothesize that HA molecules have an additional function—the function of neutralizing such an enzyme as this lysozyme. Moreover, since HA molecules, as mentioned above, are associated with the outer surface of the cell, they can form the first line of cell defense against the penetration of lysozyme molecules into the membrane. In particular, this conclusion is confirmed in earlier works [54,55] in which a noticeable decrease in the effects of the lysozymes on the membrane of Gram-negative bacteria was found by some polysaccharides.

## 4. Conclusions

The characteristics of the translational mobility obtained by NMR with PFG demonstrated the peculiarities of the interaction of HAs with bovine serum albumin (BSA) and hen egg-white lysozyme (HEWL). The characteristics of the translational mobility of HAs demonstrate the marked effects of the presence of BSA protein in the system. First, it manifests itself in the fact that the presence of BSA initiated some additional mechanism, as a result of which 100% of HA molecules formed the gel structure. In this case, the recorded decrease in the SDC value of BSA molecules because of the interaction with HAs can be interpreted because of the formation of short-lived BSA–HA complexes.

On the contrary, in the HEWL–HA system, more significant effects of protein interaction with hyaluronate are observed. Thus, the lysozyme acts as an enzyme, hydrolyzing the glycosidic bond of the polysaccharide. As a result, some of the HA molecules are degraded (torn into pieces with a lower molecular weight), in such a way that they lose the ability to form a gel structure. This effect is noticeable even at very low protein concentrations. As the protein concentration increases, the proportion of degraded HA molecules increases, but, importantly, the other part of the HA molecules retains its characteristics, including the ability to form a gel structure. The most important result, in our opinion, is the establishment of the fact that the lysozyme molecules in the process of performing the function of hydrolyzing the polysaccharide do not remain free, but form a strong complex with parts of the degraded HA molecules and thereby acquire the same characteristics of translational mobility.

With further increases in the concentration of HEWL in HA aqueous solution, the effect of HA/HEWL coacervate formation appears, due to which phase separation occurs in the system. Thus, the presence of the lysozymes in HA aqueous solution demonstrates not only the ability of HEWL to cleave the HA polymer chain, but also the ability to form intermolecular complexes with HA parts.

An important result of the interaction of lysozyme molecules with HA is the neutralization of the enzymatic activity of the lysozymes, which is probably due to the formation of the lysozyme–HA complex. Thus, HA molecules demonstrate the possibility of performing a protective function against the penetration of the lysozymes into the cell membrane.

In general, the results obtained are important enough for a deeper understanding of the mechanisms and functions of the membrane system as a whole.

## Data Availability

Data are contained within the article.
